# Sodium nitroprusside and peroxynitrite effect on hepatic DNases: an *in vitro *and *in vivo *study

**DOI:** 10.1186/1476-5926-3-6

**Published:** 2004-08-31

**Authors:** Gordana Kocic, Dusica Pavlovic, Radmila Pavlovic, Goran Nikolic, Tatjana Cvetkovic, Ivana Stojanovic, Tatjana Jevtovic, Radivoj Kocic, Dusan Sokolovic

**Affiliations:** 1 Institute of Biochemistry, Medical Faculty University of Nis, Serbia and Montenegro; 2 Institute of Chemistry, Medical Faculty University of Nis, Serbia and Montenegro; 3 Clinic for Endocrinology, Faculty of Medicine University of Nis, Serbia and Montenegro

## Abstract

**Background:**

It has been documented that nitric oxide (NO) donor sodium nitroprusside (SNP) and authentic peroxynitrite are capable of promoting apoptosis in a number of different cell types. Various endonucleases have been proposed as candidates responsible for the internucleosomal cleavage of the genomic DNA observed during apoptosis, but the main effect is attributed to the alkaline-DNases (Mg^2+^- and caspase-dependent) and acid-DNase. The aim of this study was to examine an *in vivo *and *in vitro *possibility for alkaline- and acid-DNases to be activated by SNP and peroxynitrite.

**Results:**

The effect on liver tissue alkaline and acid DNase activity together with the markers of tissue and plasma oxidative and nitrosative stress (lipid peroxidation, SH group content, carbonyl groups and nitrotyrosine formation) was investigated in plasma and liver tissue. The activity of liver alkaline DNase increased and that of acid DNase decreased after *in vivo *treatment with either SNP or peroxynitrite. A difference observed between the *in vivo *and *in vitro *effect of oxide donor (i.e., SNP) or peroxynitrite upon alkaline DNase activity existed, and it may be due to the existence of the "inducible" endonuclease. After a spectrophotometric scan analysis of purified DNA, it was documented that both SNP and peroxynitrite induce various DNA modifications (nitroguanine formation being the most important one) whereas DNA fragmentation was not significantly increased.

**Conclusion:**

Alkaline DNase activation seems to be associated with the programmed destruction of the genome, leading to the fragmentation of damaged DNA sites. Thus, the elimination of damaged cells appears to be a likely factor in prevention against mutation and carcinogenesis.

## Background

In its response to tissue damage and inflammation induced by a variety of xenobiotics, endotoxins and disease states (such as viral hepatitis), post-ischemic and regenerative injury, the liver produces a large quantity of nitric oxide (NO). Nearly all cell types in liver tissue, including hepatocytes, Kupffer cells, stellate cells and endothelial cells, have the capacity for generating NO. It has been documented that NO is capable of promoting apoptosis in a number of different cell types, generally classified as cGMP-dependent or cGMP-independent [1-4]. The potential of chemical NO donor sodium nitroprusside (SNP) to induce apoptosis directly from NO liberation has been established *in vitro *[5]. The fact that NO is capable of triggering apoptosis is consistent with its ability to induce DNA damage, the inhibition of DNA synthesis and cell cycle arrest [6,7]. The reaction product formed between NO^• ^and superoxide [i.e., peroxynitrite (ONOO^-^)] plays a critical role in the induction of inflammatory reaction and apoptosis, but is also associated with tumor promotion and/or progression. Potentially toxic levels of peroxynitrite can be achieved whenever NO^• ^and O_2_^.- ^production is stimulated, due to the fact that a 100-fold increase in the rate of peroxynitrite formation occurs for every 10-fold increase in NO^• ^and O_2_^.- ^concentration [8].

Apoptosis, frequently termed "programmed cell death", is the form of cell death that occurs in normal liver in the course of its development and organogenesis, and in adult liver during the renewal of hepatocytes. In addition, apoptosis can be triggered by several hepatotropic viruses and toxic drugs, as well as in various liver diseases and experimental liver conditions such as hepatic allograft rejection. Degradation of the nuclear DNA, a common phenomenon observed in many organisms throughout the evolutionary scale, is one of the best-characterized biochemical features of apoptotic cell death. It has been established that the cell undergoes epigenetic reprogramming in the D_1 _phase of programmed cell death, the result of which is the activation of double-stranded DNA fragmentation in the F phase during which the nuclear morphology dramatically changes [9]. The cleavage of DNA may have a protective function in that it reduces the likelihood for genes in a potentially active site to be transferred from dying cells to the nuclei of viable neighboring cells. It is possible that various endonucleases exert a DNA degrading activity, as well as that many proteins can receive DNA degrading properties upon change of pH conditions [11]. The most important aspect of apoptosis is the universal property of some proteins to exert a dual function: the protection against proteolysis and the maintenance of the structure and function of normal cells. Being free from the inhibitory complex, however, these proteins may also contribute to protein or chromatin cleavage during apoptosis [12,13]. Changes in DNA degradation may lead to the pathogenesis in various disorders, such as liver cancer [14,15].

On the basis of the pharmacological data supporting the critical role of NO and peroxynitrite in apoptosis, current research studies have evaluated the activity of alkaline and acid DNase during the administration of SNP or of peroxynitrite, as well as the changes in numerous susceptible parameters of nitrosative stress, including SH group oxidation, carbonyl group formation, lipid peroxidation and DNA modification. An assay of enzyme activity was performed using liver tissue after *in vivo *administration and *in vitro *treatment of isolated rat hepatocytes or purified commercial enzymes either with SNP or authentic peroxynitrite.

## Results

There are few data concerning the *in vivo *susceptibility of liver tissue to NO donor SNP and authentic peroxynitrite. A considerable attention has been paid to the establishment of *in vivo *tolerability and to the markers of apoptotic effects. Both NO and peroxynitrite can directly react with aromatic and sulfhydryl nucleophiles and nitrate aromatic residues. Sulfhydryl groups oxidation was documented in the plasma and, almost equally, in liver tissue. Peroxynitrite administration led to a more pronounced decrease in the concentration of other plasma free radical scavengers such as uric acid, and was followed by an increase in plasma nitrate concentration (Tables 1 and 2). According to the data suggesting that peroxynitrite decomposes rapidly to OH^• ^and NO_2 _^•^-like species at physiological pH, it was assumed that the carbonyl groups and the lipid peroxidation product [i.e., malondyaldehyde (MDA)] may play a significant role in liver cell toxicity. Neither plasma nor liver protein carbonyls showed any significant increase. This may be a likely consequence of the significant increase in aromatic amino acids nitration, presumably tyrosine the spectral contribution of which was substracted from the samples treated with 2,4-dinitrophenylhidrazine. Plasma and liver MDA concentrations were not significantly changed, either. The obtained results do not support the data suggesting that oxygen radicals, probably generated during cellular SNP metabolism, may mediate cell toxicity and apoptosis, but do confirm previous *in vitro *observations [16]. Plasma alanine aminotranferase (ALT) activity, used as the standard liver functional test, decreased in the peroxynitrite-treated group (Table 1). The activity of liver alkaline DNase increased and that of acid DNase decreased after *in vivo *treatment with either SNP or peroxynitrite (Table 2).

**Table 1:** Plasma levels of investigated parameters of Sprague-Dawley rats after *in vivo *treatment with nitric oxide donor (SNP) and peroxynitrite. Data expressed as Mean (SD); n = 8 per group.

**Parameters**	**Control**	**SNP**	**Peroxynitrite**
Lipid peroxidation (MDA) (μmol/l)	7.10 (0.86)	6.78 (2.11)	6.64 (2.85)
SH groups (μmol/l)	180.47 (36.08)	150.84 (4.78)	110.40 (11.96)*
NO_2_+NO_3 _(μmol/l)	45.47 (18.80)	66.82 (4.12)	81.94 (10.18)
Uric acid (μmol/l)	64.82 (4.18)	22.34 (8.32)	17.50 (3.21)*
Carbonyl groups (μmol/g protein)	179.59 (28.50)	144.90 (67.40)	169.06 (14.88)
Nitrotyrosine (nmol/g protein)	96.02 (26.53)	136.17 (27.47)	135.11 (4.64)
ALT (U/l)	31.9 (4.15)	34.44 (4.96)	28.5 (5.15)

Male Sprague-Dawley rats three months old were allocated into three different groups. Either peroxynitrite (0.5 ml/kg BW of 30 mmol solution) or SNP (10 mg/kg BW) in a volume of 100 μl were administrated in bolus in systemic circulation. The control group received physiological saline solution at the same volume. All procedures were carried out as described in Methods. Asterisk: significantly different from the control (p < 0.05).

**Table 2:** Liver levels of investigated parameters of Sprague-Dawley rats after *in vivo *treatment with nitric oxide donor (SNP) and peroxynitrite. Data expressed as Mean (SD); n = 8 per group.

**Parameters**	**Control**	**SNP**	**Peroxynitrite**
Lipid peroxidation (MDA) (μmol/g protein)	54.42 (8.68)	52.5 (4.34)	58.2 (6.11)
SH groups (μmol/g protein)	636.04 (74.58)	538.67 (82.87)	505.52 (41.43)
NO_2_+NO_3 _(μmol/g protein)	6.15 (2.91)	6.04 (2.61)	5.74 (1.03)
Carbonyl groups (μmol/g protein)	4.14 (0.13)	4.25 (0.12)	4.20 (0.26)
Nitrotyrosine (nmol/g protein)	10.26 (2.62)	11.40 (1.24)	16.62 (3.58)
DNA (mg/g tissue)	10.71 (1.75)	12.65 (1.01)	12.15 (1.25)
DNA fragmentation (%)	11.99 (0.33)	13.21 (1.43)	13.0 (0.89)
Alkaline-DNase (U/g protein)	11.95 (0.57)	15.30 (0.72)	16.45 (1.04)
Acid-DNase (U/g protein)	15.28 (3.42)	12.34 (0.35)	9.34 (0.76)

Male Sprague-Dawley rats three months old were allocated into three different groups. Either peroxynitrite (0.5 ml/kg BW of 30 mmol solution) or SNP (10 mg/kg BW) in a volume of 100 μl were administrated *in bolus *in systemic circulation. The control group received physiological saline solution at the same volume. All procedures were carried out as described in Methods.

**Figure 1:**
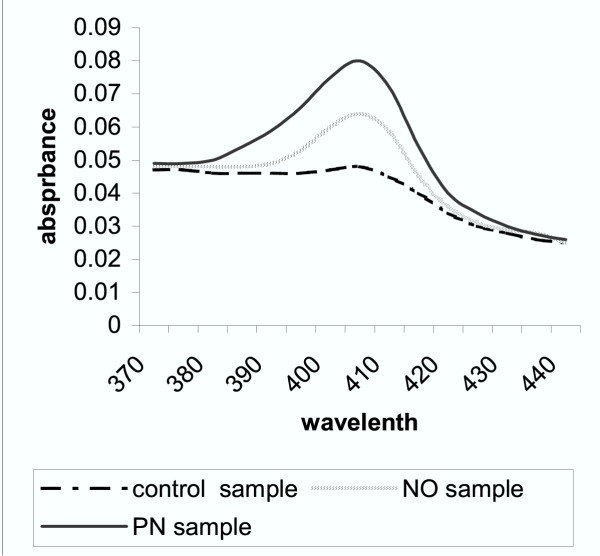
**The peak appearance of isolated liver DNA**. The extraction oftissue DNA was performed according to the method of Wannemacher et al. [50], modified by Setaro & Morley [51], with the protein and nucleic acid precipitation by using ice-cold trichloroacetic acid after lipid extraction. DNA was separated from proteins by hydrolisis of resulting pellet at 96 ± 1°C for 45 min. Samples were analyzed for DNA concentration by ultraviolet absorption difference at 260 and 290 nm. Purified DNA was employed for spectral changes, monitored by using Beckman spectrophotometer. On the basis of the data obtained by Yermilov et al. [13], the appearance of a peak between 375 and 405 nm (depending on pH) corresponds to 8-nitroguanine. The peak appearance was between 390 and 410 nm with the maximum absorbance at 405 nm, obtained in alkaline conditions (DNA extract was adjusted to pH 10).

The ultraviolet spectra of DNA were obtained by spectrophotometric scanning between 230–500 nm on a scan detecting system. According to the data by Yermilov et al. [17], the appearance of a peak between 375 and 405 nm (depending on pH) corresponds to 8-nitroguanine. In the scan analysis (Figure 1), the peak was between 390 and 410 nm with a maximum absorbance at 405 nm in alkaline conditions (DNA extract was adjusted to pH 10). This peak may correspond to the formation of nitro-derivatives, most probably of 8-nitroguanine. The nitroguanine peak at 405 nm was particularly apparent in peroxynitrite-treated samples.

*In vivo *administration of SNP or peroxynitrite tended to increase the rate of DNA fragmentation, but it was not statistically significant. The rate was estimated according to the percentage of DNA resisting centrifugation at 27 000 g (Table 2).

**Figure 2:**
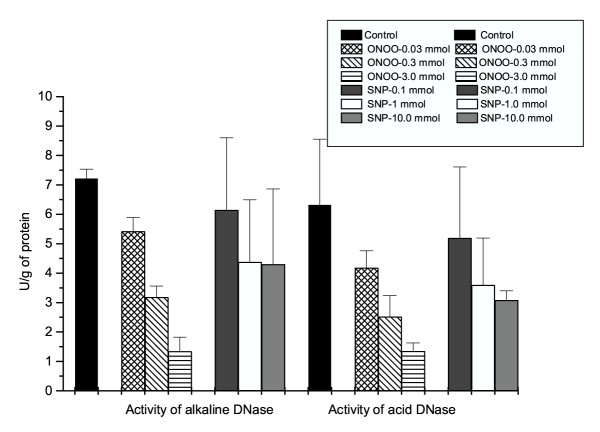
**The activity of alkaline and acid DNase after *in vitro *treatment of isolated hepatocytes with NO donor (SNP) or peroxynitrite**. The isolation of hepatocytes was done according to the method already published [42], by using a 1% collagenase dissolved in RPMI 1640 medium. Hepatocytes, isolated from 8 Male Sprague-Dawley rats, were dissolved in a physiological saline solution in a concentration of approximately 10^8^cells/ml. They were divided into seven groups (each comprising 8 samples), exposed to either SNP (0.1, 1 and 10 mmol) or peroxynitrite (0.03, 0.3 and 3 mmol) for a period of 1 hour at 37°C. Given *in vitro *concentrations were calculated according to the literature data [38]. The activity of alkaline and acid-DNase was measured by the methods of Bartholeyns et al. [43] and acid soluble nucleotides were determined spectrophotometrically at 260 nm. The enzyme activity was expressed as U/g protein. Data (n = 8) in graph is putted as: Mean + SD.

**Figure 3:**
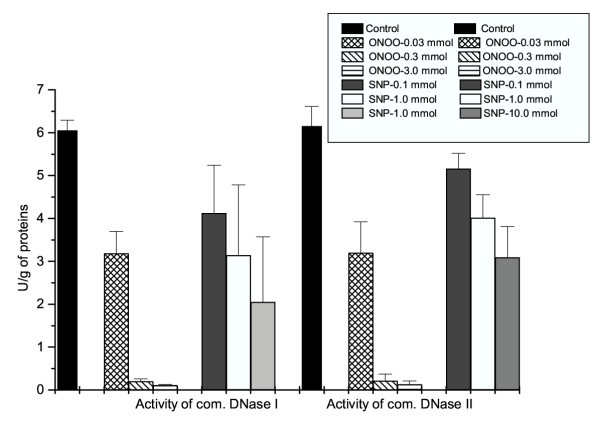
**The activity of commercial DNase I and DNase II after in vitro treatment with NO donor (SNP) or peroxynitrite. **Purified enzymes DNase I (E.C. 3.1.21.1) and DNase II (E.C. 3.1.22.1) were dissolved in physiological saline solution. Hepatocytes, isolated from 8 Male Sprague-Dawley rats, were dissolved in a physiological saline solution in a concentration of approximately 10^8 ^cells/ml. They were divided into seven groups (each comprising 8 samples) exposed to either SNP (0.1, 1 and 10 mmol) or peroxynitrite (0.03, 0.3 and 3 mmol). The reducing agent (cysteine 1 mmol) was added to SNP to induce *in vitro *NO release [39]. The activity of alkaline and acid-DNase was measured by the methods of Bartholeyns et al. [43] and acid soluble nucleotides were determined spectrophotometrically at 260 nm. The defined units for purified DNase I and DNase II (increase in absorbance of 0.001/min in a sample containing 0.132 mg DNA, pH 7.4 or pH 5 and 3 ml of reaction mixture) were obtained from the Sigma catalogue label. Data (n = 8) in graph is putted as: Mean + SD.

After *in vitro *exposure of isolated hepatocytes to SNP or peroxynitrite, the activity of both alkaline and acid DNase decreased in a dose-dependent fashion (Figure 2). During *in vitro *incubation of purified enzymes DNase I and DNase II with SNP or peroxynitrite, a dose-dependent decrease of enzyme activity was also documented (Figure 3).

## Discussion

NO^•^, a free radical gaseous molecule is one of the simplest compounds found to be continuously produced in humans and animals. It can be derived from L-arginine through the enzyme nitric oxide synthase (NOS) and by different NO donors, including SNP. NO has been shown to play an unprecedented range of roles in biological systems, acting as a universal intracellular and transcellular signaling molecule and the regulator of vascular tone, cell proliferation and apoptosis [18-20]. Peroxynitrite is a strong, relatively long-lived oxidant with a half-life of approximately 0.5–1 s under physiological conditions. Our study confirmed that in both plasma and liver tissue peroxynitrite causes a rapid oxidation of sulfhydryl groups and thioethers, as well as the nitration and hydroxylation of aromatic compounds (Tables 1 and 2). A chronic exposure of hepatocytes to reactive nitrogen species exhibits a cytotoxic and cytostatic activity leading to functional and morphological alterations [8,21]. Cell death after exposure to different NO-donors such as SNP has been to date established through the expression of tumor suppressor gene p53 and pro-apoptotic genes such as bax, cyclin-dependent kinase inhibitor p21, the inhibited expression of anti-apoptotic protein bcl-2, the inhibited NF-κB binding activity, ERK and p-38-dependent cytochrome c release, and caspase-3 activation [22-24]. In contrast, the anti-apoptotic effects of NO may be mediated through the mechanisms such as blockade of the recruitment of pro-caspase-9 to the Apaf-1 apoptosome, stimulation of c-GMP-dependent protein kinase, control of mitochondrial permeability transition, induction of the heat shock protein HSP 70, and interaction with the ceramide pathway [25,26]. The prolonged damage of p53 gene by peroxynitrite has been associated with tumor formation. Recent results by Vincent and Maiese [3] indicate that NO donor SNP (at 300 μmol concentration) is capable of inducing strong apoptotic effects via DNA fragmentation and induction of Mg^2+^-dependent endonuclease activity in the culture of neuronal cells. In our *in vivo *study (Table 2), the activity of alkaline DNase increased within 24 h after exposure to SNP (achieving approximately a similar blood concentration of about 250 μmol) or to authentic peroxynitrite. Several molecules involved in nuclear DNA fragmentation have been detected and characterized based on their ionic sensitivity. Besides the presence of constitutive Ca^2+^/Mg^2+^-dependent endonucleases, a great deal of endonuclease activity within a 7.2–8.0 pH range most probably represents the inducible form of DNase. The molecular weights of the constitutive (NO-independent) and inducible (NO-dependent) endonuclease are similar, as well as their optimum pH range (7.5–8.0). A likely conclusion is that Mg^2+^-dependent endonuclease seems to be a result of *de novo *synthesized or the pre-existing Ca^2+^/Mg^2+^-dependent endonuclease activation. Up to now, several Mg^2+^- or Ca^2+^/Mg^2+^-dependent alkaline DNases (DNase I) with an optimum activity within the range of 7.5–9.5 have been purified. Some of them, including specific caspase3-activated DNase (CAD), are active upon release of the specific inhibitor ICAD [27,28]. DNase gamma has been documented as a critical component of apoptotic machinery, in that it cleaves the chromosomal DNA into nucleosomal units, thus leading to DNA ladder formation [29]. The alkaline DNase, active only during apoptosis, has been documented to be inherent to cyclophilins (A, B and C) as well, irrespective of their protein folding (peptidylprolyl cis-trans-isomerase) activity. All of them have the ability to degrade the supercoiled, single stranded and double stranded DNA [30,31]. Besides alkaline DNases, the cation-independent endonuclease with an optimum activity at pH 5, known as acid or DNase II, was identified. One leucocyte elastase inhibitor (LEI) can also exert an acid DNase activity after post-translational modification through the proteolytic cleavage [32]. The specific involvement of DNase II in physiological nuclear degradation during apoptosis could not be excluded upon decrease of intracellular pH values below 7 with a proton ionophore. Three potential *N*-nitrosylation sites are important for DNase II regulation [32,33]. Since our experimental data indicated a decrease in acid DNase activity 24 h after exposure to SNP or peroxynitrite (Table 2), the inhibition of DNase II may be explained by the nitrosylation of its susceptible sites. Indeed, when isolated hepatocytes were exposed to SNP or peroxynitrite for 1 h, a dose-dependent inhibition of DNase II was also documented (Fig. 2). The same result was obtained after exposure of purified enzyme to SNP (in the presence of the reducing agent cysteine 1 mmol) or peroxynitrite (Fig. 3).

The formation of 8-nitroguanine, 8-oxo-deoxyguanine and oxazolone and the oxidative modification of 2'-deoxyribose into TBA-responsive compounds are the most prominent nucleotide modifications after reactive nitrogen species attack [34,35]. A highly potential mutagenic product 8-nitroguanine can be depurinated yielding apurinic sites capable of inducing GC→TA transversions, GC→CG transversions and deletions [17,36]. The appearance of the nitroguanine peak during the scan analysis of purified DNA at 405 nm was documented in our study (Fig. 1). The rate of DNA fragmentation tended to be increased, but the difference was not significant (Table 2).

## Conclusions

*In vivo *administrated SNP and peroxynitrite increase the activity of alkaline DNase. They also induced DNA modifications, such as nitroguanine formation. The obtained DNase activation seems to be associated with the programmed destruction of the genome and cell death. Given the above results and observations, the elimination of damaged hepatic cells appears to be a likely factor in prevention against mutation and carcinogenesis.

## Methods

### Chemicals

SNP, DNA, DNase I (E.C. 3.1.21.1.) and DNase II (E.C. 3.1.22.1) were obtained from Sigma-Aldrich Company. RPMI-1640, fetal calf serum (FCS) and collagenase were purchased from ICN (Costa Mesa, CA). Authentic peroxynitrite was freshly synthesized by the quench-flow technique [37] and its concentration was monitored in alkaline solution before use in each experiment by measuring the extinction coefficient at 302 nm [38]. All other chemicals were of the highest purity range.

### *In vivo *study

Twenty-four male Sprague-Dawley rats, three months old, were divided into three different groups, each comprising 8 animals. Either SNP (10 mg/kg BW) or peroxynitrite (0.5 ml/kg BW of 30 mmol solution) in a volume of 100 μl were administrated *in bolus *in systemic circulation by intraventricular injection under penthobarbital sodium anesthesia. The concentrations were calculated according to the literature data concerning their *in vivo *tolerability and in vitro ability to induce apoptotic effects [39,40]. The calculation of peroxynitrite intra-arterial concentration (6 nmol) was done according to its biological half-life of about 0.6 s, cardiac output of 40 ml/min/100 g and circulating volume of 20 ml and 250 g of rat BW [41]. The corresponding control group received physiological saline solution in the same volume. The rats were killed 24 h afterwards, under the same anesthesia. Blood was collected from the abdominal aorta and livers were quickly removed, frozen and homogenised on ice.

### Isolation of hepatocytes

The isolation of hepatocytes was done according to a method already published [42], by using a 1% collagenase dissolved in RPMI 1640 medium. Collagenase was inhibited by using 10% FCS and cells were washed twice in physiological saline solution. Hepatocytes were isolated from 8 Male Sprague-Dawley rats. They were dissolved in a physiological saline solution in a concentration approximately 10^8 ^cells/ml. They were divided into seven groups (each comprising 8 samples), exposed to either SNP (0.1, 1 and 10 mmol) or peroxynitrite (0.03, 0.3 and 3 mmol) for a period of 1 hour at 37°C. Given *in vitro *concentrations were calculated according to the literature data [38]. Purified enzymes DNase I and DNase II were dissolved in physiological saline solution, exposed to the same concentrations of SNP and peroxynitrite, except that the reducing agent (cysteine 1 mmol) was added to SNP to induce *in vitro *NO release [39].

### Methods for alkaline and acid-DNase

The activity of alkaline and acid-DNase was measured by the methods of Bartholeyns et al. [43] and acid soluble nucleotides were determined spectrophotometrically at 260 nm. The enzyme activity was expressed as U/g protein, for tissue and cell samples. The defined units for purified DNase I and DNase II (increase in absorbance of 0.001 / min in a sample containing 0.132 mg DNA, pH 7.4 or pH 5 and 3 ml of reaction mixture) were obtained from the Sigma catalogue label.

### Extraction of DNA and proteins

The extraction of tissue DNA and proteins was performed according to the method of Wannemacher et al. [44] modified by Setaro & Morley [45] by protein and nucleic acid precipitation using ice-cold trichloroacetic acid (TCA), 0.6 N, after lipid extraction. RNA and DNA were isolated by using cold 60% perchloric acid (PCA). DNA was separated from proteins by hydrolysis of resulting pellet at 96 ± 1°C for 45 min after adding 0.5 N PCA. Tissue protein content was measured according to the Lowry et al. procedure [46]. Samples were analysed for DNA concentration by an ultraviolet absorption difference at 260 and 290 nm. Purified DNA was employed for spectral changes monitored by using Beckman DU 530 spectrophotometer. Protein carbonyls and protein nitrotyrosine were measured in plasma proteins and the remaining protein pellet according to the method of Oliver et al. [47] modified by Tien et al. [48]. DNA fragmentation assay was performed according to the method of Jones et al. [49] based on the percentage of DNA resisting centrifugation at 27 000 g for 20 min. The proportion is expressed as percentage of the total DNA in the uncentrifugated sample. Protein carbonyls were quantified by spectrophotometric measurement of their 2,4 dinitrophenylhydrazone derivatives (ε 370 nm = 22000 M-1 cm^-1^). The difference between the spectrum of the DNPH-treated sample and that of the HCl control was determined and expressed as μmol DNPH/g protein. As nitrotyrosine also absorbs at 370 nm, it was measured according to its spectral contribution at 370 nm. Plasma and tissue SH groups were measured by using DTMB according to the Elman method [50]. Plasma and tissue lipid peroxidation product MDA was measured according to the method of Ohkava et al. [51]. Nitrates were measured according to the method of Navarro-Gonzales et al. [52]. Plasma uric acid and ALT were measured using the Synchron analyzer.

### Statistics

Statistical analysis was made with the software SPSS. The effect of treatments was firstly evaluated by one-way ANOVA. If there was a significant effect, experimental data sets were compared against the control group by the Dunnett post hoc test. Significance level was set at α = 0.05. Data were normally distributed with equal variances among groups.

## Authors' contributions

GK carried out the *in vivo *and *in vitro *experiments, culture experiments and wrote the paper. RP and GN carried out DNA spectral analysis. TC performed measurement of SH groups and lipid peroxides. IS performed the measurement of nitrates and nitrites. TJ assisted during *in vivo *and *in vitro *experiments and did the graphical presentation. DS performed statistical analysis and assisted during *in vivo *experiments. DP and RK assisted during *in vitro *experiments and participated in the design of the study. All the authors read and approved the final manuscript.
